# The multiple roles of sucrase-isomaltase in the intestinal physiology

**DOI:** 10.1186/s40348-016-0033-y

**Published:** 2016-01-26

**Authors:** Birthe Gericke, Mahdi Amiri, Hassan Y. Naim

**Affiliations:** Department of Physiological Chemistry, University of Veterinary Medicine Hannover, Buenteweg 17, 30559 Hannover, Germany

**Keywords:** Sucrase-isomaltase, Congenital sucrase-isomaltase deficiency, Carbohydrate maldigestion, Gastrointestinal intolerance, Inflammation, Gut microbiota

## Abstract

Osmotic diarrhea and abdominal pain in humans are oftentimes associated with carbohydrate malabsorption in the small intestine due to loss of function of microvillar disaccharidases. Disaccharidases are crucial for the digestion and the subsequent absorption of carbohydrates. This review focuses on sucrase-isomaltase as the most abundant intestinal disaccharidase and the primary or induced pathological conditions that affect its physiological function. Congenital defects are primary factors which directly influence the transport and function of sucrase-isomaltase in a healthy epithelium. Based on the mutation type and the pattern of inheritance, a mutation in the *sucrase*-*isomaltase* gene may exert a variety of symptoms ranging from mild to severe. However, structure and function of wild type sucrase-isomaltase can be also affected by secondary factors which influence its structure and function either specifically via certain inhibitors and therapeutic agents or generally as a part of intestinal pathogenesis, for example in the inflammatory responses. Diagnosis of sucrase-isomaltase deficiency and discriminating it from other gastrointestinal intolerances can be latent in the patients because of common symptoms observed in all of these cases.

Here, we summarize the disorders that implicate the digestive function of sucrase-isomaltase as well as the diagnostic and therapeutic strategies utilized to restore normal intestinal function.

## Dietary carbohydrates as human energy source

Carbohydrates are one of the vital mammalian nutrients which have been increasingly consumed by human populations since the agricultural revolution and serve as important calorie sources [1]. Dietary carbohydrates mainly include sucrose and a variety of plant starches which are composed of different α-linked sugars. Sugar transporters in the intestine are only capable of transporting monosaccharides. Therefore, accessibility of higher carbohydrates to the human body as energy sources necessitates their hydrolysis to simple monosaccharides. Starch digestion is initiated by salivary and later pancreatic α-amylases that break it down to smaller units composed of two, three, or four sugar residues. The final step of starch digestion takes place in the intestinal lumen by α-glucosidases that are localized on the brush border membrane (BBM) of the intestinal epithelium. This family of digestive enzymes includes sucrase-isomaltase (SI), maltase-glucoamylase (MGAM) and trehalase [2]. Due to its high abundance and its wide substrate specificity in hydrolyzing α-1,2, α-1,6, and α-1,4 glucosidic bonds, human SI is responsible for almost all sucrase activity and about 60 to 80 % of maltase activity in the intestinal lumen [3].

## Physiological and functional requirements for carbohydrate processing

Before SI can fulfill its hydrolytic function in the intestinal lumen, it needs to be intracellularly processed and properly transported to the BBM surface of the epithelial cells. Both active subunits are then oriented into the intestinal lumen [4]. SI is a type II membrane glycoprotein. It is N-glycosylated in the endoplasmic reticulum (ER) and then transported to the Golgi apparatus where it is complex N- and O-glycosylated. Both of these modifications have a crucial role in the folding, acquisition of the functional capacity, and later appropriate sorting of the SI molecules to the BBM [5]. The latter process is mediated via association of SI molecules with cholesterol- and sphingolipid-enriched membrane microdomains, known as lipid rafts [6], in the *trans*-Golgi network for which proper O-glycosylation is essentially required [5].

## Etiology and common symptoms of intestinal disorders by SI malfunction

The clinical presentation of intestinal disorders caused by SI malfunction is oftentimes an osmotic-fermentative diarrhea upon ingestion of carbohydrates associated with symptoms such as vomiting, flatulence and abdominal pain [2]. An increased osmotic load due to unabsorbed carbohydrates in the intestinal lumen and colon causes a flow of water and electrolytes into the lumen, thus affecting the gut motility by accelerating the small intestinal transit leading to chronic diarrhea. Other symptoms like gaseous abdominal distension, flatulence, and cramps are caused by bacterial fermentation of unabsorbed carbohydrates which additionally increase the osmotic pressure [3]. The accelerated transit further increases the degree of maldigestion of starch and monosaccharides and also affects the absorption of other nutrients, leading to a risk of malnutrition and failure to thrive in patients [7]. Additional factors contributing to the onset of disease are the quantity of ingested carbohydrates and other food components, the rate of gastric emptying, the metabolic activity of fermenting bacteria, the degree of the residual enzyme activity of SI and other intestinal disaccharidases as well as coexisting intestinal disorders or inflammation.

SI deficiencies can be categorized as (a) primary or congenital SI deficiencies which are caused by genetic alterations in the *SI* gene and are already present at birth and (b) induced or secondary SI deficiencies which occur later in life and are mediated by environmental factors or arise as a consequence of other genetic deficiencies which negatively influence the intestinal physiology in general and SI function in particular.

## Congenital sucrase-isomaltase deficiency

Congenital sucrase-isomaltase deficiency (CSID) is a genetically determined primary defect of SI which is associated with carbohydrate malabsorption [3]. CSID is elicited by single-nucleotide polymorphism in the *SI* gene leading to single amino acid exchanges in the protein. These mutations can affect each of the SI domains but do not *per se* lead to complete lack of SI or its activities in the BBM essentially. Requirements for proper functioning of SI, including intact glycosylation and folding, correct intracellular trafficking, association with lipid rafts and apical delivery to the cell surface are directly or indirectly correlated to each other and are initially regulated at the amino acid sequence level [5]. Malfolding of SI mutants may affect the trafficking or even lead to degradation via the endoplasmic reticulum-associated protein degradation (ERAD) pathway [8]. Other mutants of SI proteins with impaired or delayed trafficking though with normal hydrolytic properties cannot fulfill their physiological functions due to mislocalization. Mutations can affect one or more of these features depending on their position within SI. Biochemical, cellular and functional analyses of a number of SI mutations constituted the basis for a classification of these mutants into seven phenotypes depending on the intracellular localization and function of SI [9] (Table 1). Phenotypes I and II include mutations which are blocked in the ER (I) or ER-Golgi intermediate compartment (ERGIC) and *cis*-Golgi compartments (II). A normal trafficking but absent enzymatic function characterizes mutations of phenotype III. SI mutants grouped in phenotype IV exhibit sorting defects to the microvillar membranes and that of phenotypes V and VI are intracellularly cleaved. Phenotype VII is characterized by altered folding, increased turnover rate, and partial missorting [9]. The most common mutations with an estimated frequency of 83 % in European descendants with CSID are Gly1073Asp, Val577Gly, Phe1745Cys and Arg1124Stop [10]. These mutations are intracellularly blocked in the ER and belong to phenotype I. A decisive factor in occurrence and severity of CSID is whether only one or both alleles of the gene are affected by mutations. Initially reported cases of CSID that were characterized by genetic sequencing mostly illustrated homozygote or compound heterozygote patterns of inheritance [11]. In the recent years, feasibility of gene sequencing has led to identification of many novel cases of CSID, among which also simple heterozygote subjects with only one affected allele exist [10].

**Table 1:** Phenotypic categorization of SI mutants

Phenotype of SI mutants	Transport and function
I	Blocked in the ER, enzymatically inactive
II	Blocked in the ER, ER-Golgi intermediate compartment, and *cis*-Golgi, inactive
III	Normal intracellular protein transport but absent activities
IV	Sorting defects, enzymatically active
V	Intracellular proteolytic cleavage and degradation of sucrase, normal transport and partial activity
VI	Intracellular proteolytic cleavage and secretion of SI, complete activity
VII	Altered trafficking and increased turnover rate, partially active

Based on the mutation type, the combinatorial effects of two mutations or of one mutation with the wild-type protein can set the course of the disease and generate a mild to severe gradient of symptoms. The CSID symptoms are similar to those of other intestinal diseases and oftentimes lead to misdiagnosis or late diagnosis after childhood [3]. The high phenotypic diversity, genetic heterogeneity and common symptoms with other intestinal diseases lead to the assumption that CSID is more common than what has been initially estimated.

## Secondary deficiencies of SI activity

The major types of secondary or induced SI deficiencies can be categorized into three main groups: (a) those that are induced by physical injuries to the intestine and disrupt the intestinal epithelium, (b) those that are caused via inhibitory function of some dietary components or therapeutic agents on the function of SI, and (c) those that are connected to infections or autoimmune disorders. In what follows, we will discuss the role of therapeutic agents and autoimmune disorders on SI deficiency in more details.

i) Therapeutic agents that inhibit the SI function

There exist a large number of reports on the natural or synthetic compounds for deliberate reduction of disaccharidase activities in the intestine in order to control obesity or diabetic conditions. Apart from such compounds, our focus here is on medications that induce SI inhibition as an adverse effect. For example, *N*-butyldeoxynojirimycin (miglustat) that is used for treatment of lysosomal storage diseases can heavily inhibit SI and thus result in gastrointestinal symptoms in the majority of the patients [12]. Codeine as a pain medication and ranitidine with antihistamine effect can also inhibit sucrase activity in the intestine [13, 14]. Furthermore, several herbal folk remedies, especially those with polyphenolic components, also exert an inhibitory effect on SI [15].

ii) Role of gut autoimmune disorders in SI deficiency

Intestinal lymphatic tissue, named gut-associated lymphoid tissue (GALT), is known to be the largest compartment of the immune system. The intestinal tract is highly and directly exposed to foreign material through the ingestion of food. On the other hand, the commensal bacteria in the colon, the gut microbiota, can be differently modulated by diet or physiological conditions of the gut and can consequently influence the gut immune system [16]. All of these factors have rendered the gut tissue highly susceptible to autoimmune disorders and the subsequent tissue pathogenesis.

iii) Celiac disease

Celiac disease (CD) is one of the most common causes of chronic intestinal malabsorption and leads to occurrence of conventional gastrointestinal symptoms as a result of villus atrophy [17]. The disease is an autoimmune disorder that is triggered by hypersensitivity to ingested gliadins from wheat and other cereals [18]. The frequency of this disease can be up to 3 % in the different populations, but this ratio was detected to be as high as 11 % among patients with type 1 diabetes mellitus [19]. As a direct consequence of villus atrophy in CD, the expression and activity levels of intestinal disaccharidases including SI are significantly reduced. Therefore, SI expression has been used as an indicator to diagnose and later evaluate the physiological response to different treatments of the patients [20, 21]. Disaccharidase deficiencies can be also detected in CD patients with intact villi, which may represent latent development of CD [22].

iv) Inflammatory bowel disease

Inflammatory bowel disease (IBD) which is mostly diagnosed in the forms of Crohn’s disease and ulcerative colitis is an autoimmune disease in which imbalance between anti- and pro-inflammatory cytokines results in severe organ pathology and loss of barrier function in the intestinal tissue [23]. Induced colitis in animal models revealed local colon inflammation affecting the intestinal function in the ileal and jejunal areas and resulting in loss of SI expression and activity [24]. Besides that, the existing SI molecules are not properly expressed at the apical cell surface and reveal a predominant intracellular localization [25]. In a Caco-2 cell model of the intestinal epithelium, treatment with interleukin 6 and interferon gamma led to a significant decrease in the biosynthesis of SI [26]. Therefore, it can be concluded that SI deficiency in IBD patients occurs as an indirect effect of tissue injury alongside the direct effects of the inflammatory cytokines on the expression and function of SI.

v) Human immunodeficiency virus infection

Gastrointestinal intolerances are common symptoms in human immunodeficiency virus (HIV)-infected patients [27]. In contrast to healthy HIV-positive individuals, severe SI and lactase-phlorizin hydrolase deficiencies are identified in most of the jejunal specimens from patients with acquired immune deficiency syndrome (AIDS) [27]. The gastrointestinal symptoms are associated with rapid small bowel transit [28] and are not influenced by anti-retroviral therapy [29]. Infection of rhesus monkeys with simian immunodeficiency virus (SIV) is a known experimental animal model for HIV. As shown in this model, gut-associated lymphoid tissue is one of the first targets of SIV infection [30]. Negative regulation of the IL-6-STAT3 pathway seems to be responsible for the persistent inflammation in the jejunum and colon of these animals [31]. Development of disaccharidase deficiencies in later stages of HIV infection can be also associated with immunodeficiency or infection with opportunistic enteropathogens [30].

vi) Giardiasis

Giardiasis can also trigger autoimmune responses that cause mucosal alterations in the intestine. In this case, the microvilli are shortened and the level of SI as well as other disaccharidases is reduced in a direct relation to the number of parasites [32]. In a study using isolated T cells from infected mice, Scott et al. could show that the CD8(+) T cells mediate the loss of brush border area and reduce disaccharidase levels in the uninfected recipient mice [33].

vii) Other secondary factors influencing the SI function

Additionally reported factors influencing the function of SI and thereby triggering carbohydrate malabsorption symptoms are summarized in this part. Rotavirus infections or acute *Yersinia enterocolitica* infections affect the microvillar cytoskeleton structure of the intestinal enterocytes and thereby cause a decreased disaccharidase expression and function at the BBM [34, 35]. Also, the Shiga toxin (Stx) is reported to affect SI function by inhibiting the synthesis of BBM proteins as well as damaging the intestinal epithelium directly [36]. If the intestinal tissue is subjected to ischemia with blocked or decreased blood flow, a decrease in disaccharidase activities can be detected [37]. Chronic psychological stresses as well as deficiencies of iron and vitamin A are other factors that are associated with loss of the sucrase activity in the intestine [38–41].

## Diagnosis

The gold standard for the diagnosis of intestinal disorders associated with carbohydrate malabsorption due to primary or secondary defects in SI are endoscopic small bowel biopsies and assessment of disaccharidase activities exvivo. The additional histological examination of the intestinal biopsy specimen allows distinguishing between primary and secondary disaccharidase disorders [42]. In primary disorders, normal intestinal morphology concomitant with reduced enzymatic activities are expected while secondary disorders oftentimes go along with an impaired intestinal morphology. Confocal laser endomicroscopy (CLE) is a novel endoscopic technique to visualize the intestinal morphology in vivo [43]. A non-invasive but not always reliable diagnostic approach is the hydrogen breath test that measures the exhaled H_2_ levels produced by bacterial fermentation after ingestion of a test carbohydrate. An increased hydrogen of more than 20 part per million is considered to indicate carbohydrate malabsorption [44]. Mutations in the *SI* gene causing CSID can be identified by genetic screenings using saliva or blood of patients. Prior to the mentioned diagnostic methods, a dietary assessment by food exclusion and observation of symptom improvement can give first hints to carbohydrate malabsorption. Blood and fecal tests can additionally provide information on possible intestinal inflammation [45].

## Therapy

There are different treatment options of disaccharide malabsorption due to SI malfunction. One possibility is dietary management by a life-long sucrose- and starch-restricted diet adapted to the requirements of the patient. A good alternative to the avoidance of malabsorbed sugars, which may not be easy especially for children, is the enzyme replacement therapy to compensate the malfunction of SI. Sacrosidase or invertase (Sucraid) which is a liquid preparation from *Saccharomyces cerevisiae* is frequently used as therapy for intestinal disorders associated with carbohydrate malabsorption [3]. Prevention and treatment of secondary intestinal disorders with indirect influence on the SI function can be achieved by the ingestion of probiotics which are beneficial microorganisms to the human health [46]. The normal intestinal flora or microbiota provides an effective barrier against pathogenic microorganism. Disruption of this healthy microflora can increase the susceptibility for pathogenic infections which may in turn affect the BBM and thereby negatively influence the SI function. Intake of prebiotics (poorly digestible carbohydrates) and probiotics supports the maintenance or reestablishment of a healthy gut microflora [46, 47].

## Conclusions

**Fig. 1:**
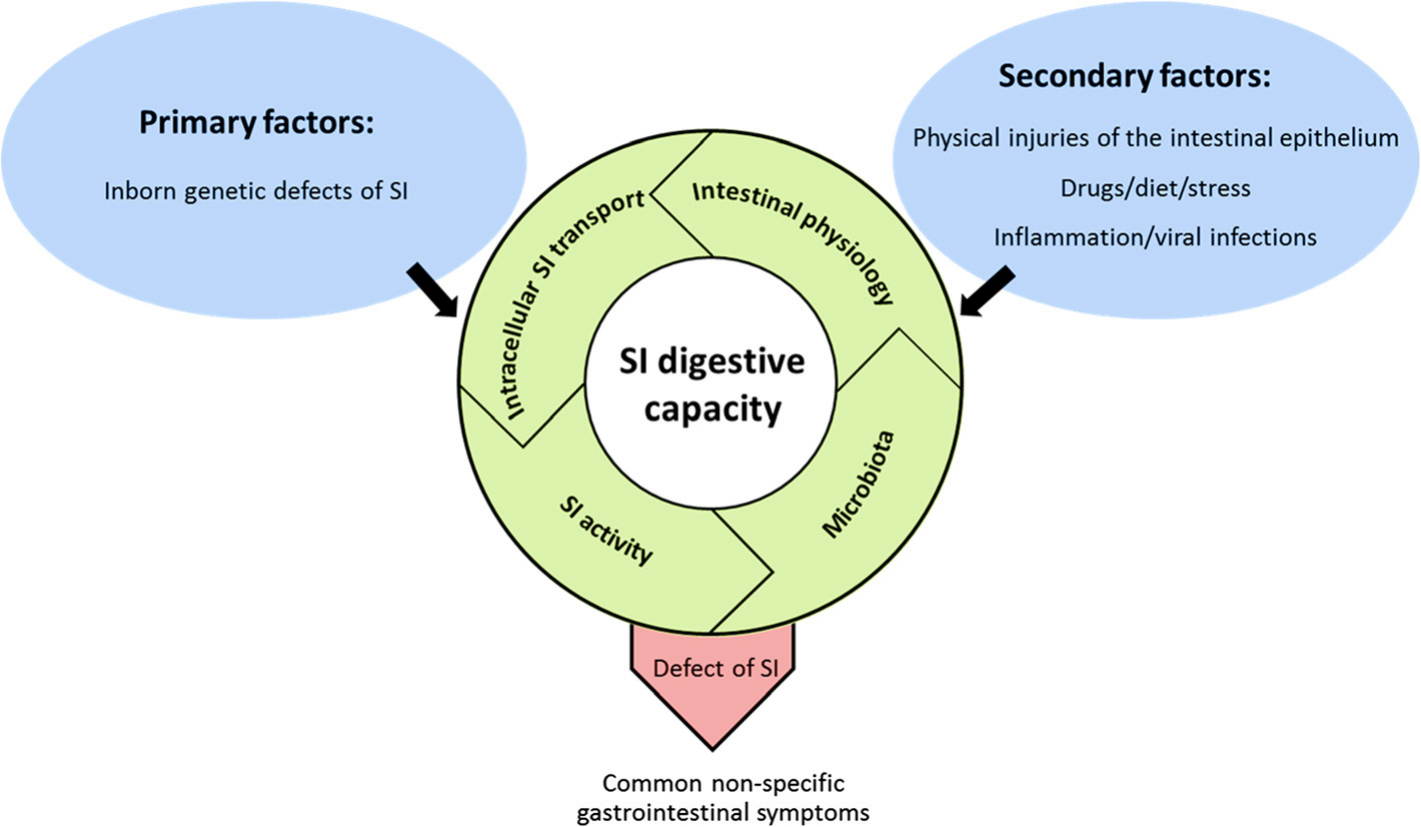
Multifactorial causes of primary or secondary sucrase-isomaltase deficiencies. The SI function in cleaving carbohydrates depends on the correct transport of the enzymatically competent protein to the BBM. This process occurs via association of SI with lipid rafts in the *trans*-Golgi network vesicles that are transported to the BBM along the actin cytoskeleton. The digestive capacity of SI can be affected by primary genetic mutations that target the *SI* gene. On the other hand, environmental factors can also influence expression and function of SI. The induced or secondary effects on the SI function can be elicited by altered epithelial cell integrity or the cytoskeletal organization of the enterocytes as well as changes in the healthy protective microflora. In some cases, the structure and function of SI can be directly influenced by certain therapeutics or dietary components. Together, alterations in the intestinal physiology triggered for example by inflammation can exert adverse effects on the trafficking and function of SI. The maldigested carbohydrates will in turn moderate the gut microbiota differently from healthy conditions and may render subsequent negative influences on the intestinal physiology

SI is a unique enzyme of the intestinal epithelium due to its high prevalence and its wide substrate specificity for digestion of different dietary carbohydrates. Therefore, deficiencies in the SI function can substantially disrupt the intestinal physiology, a fact associated with gastrointestinal symptoms, weight loss, and immunological disorders mediated by altered gut microbiota. On the other hand, organ pathologies that generally influence the intestinal tissue, such as IBD or specific inhibition of SI by therapeutic or dietary substances can substantially reduce the capacity of the intestinal lumen in SI activity (Fig. 1). If not treated, the outcome of this condition can result in progressive and life-threatening gastrointestinal symptoms. Therefore, it is legitimate to consider a major role for the proper function of SI in the maintenance of the intestinal physiology.

## Abbreviations

AIDS: acquired immune deficiency syndrome
BBM: brush border membrane
CD: celiac disease
CLE: confocal laser endomicroscopy
CSID: congenital sucrase-isomaltase deficiency
ER: endoplasmic reticulum
ERAD: endoplasmic reticulum-associated protein degradation
ERGIC: ER-Golgi intermediate compartment
GALT: gut-associated lymphoid tissue
HIV: human immunodeficiency virus
IBD: inflammatory bowel disease
MGAM: maltase-glucoamylase
SI: sucrase-isomaltase
SIV: simian immunodeficiency virus
Stx: Shiga toxin
